# Immunomodulatory and Antimicrobial Activity of Babassu Mesocarp Improves the Survival in Lethal Sepsis

**DOI:** 10.1155/2016/2859652

**Published:** 2016-08-18

**Authors:** Elizabeth S. B. Barroqueiro, Dayanna S. Prado, Priscila S. Barcellos, Tonicley A. Silva, Wanderson S. Pereira, Lucilene A. Silva, Márcia C. G. Maciel, Rodrigo B. Barroqueiro, Flávia R. F. Nascimento, Azizedite G. Gonçalves, Rosane N. M. Guerra

**Affiliations:** Centro de Ciências Biológicas e da Saúde, Laboratorio de Imunofisiologia, Universidade Federal do Maranhão, Cidade Univesitária Dom Delgado, No. 1966, Avenida dos Portugueses, 6080-580 São Luís, MA, Brazil

## Abstract

*Attalea speciosa* syn* Orbignya phalerata* Mart. (babassu) has been used in the treatment of inflammatory and infectious diseases.* Aim of the study*. To investigate the antimicrobial and immunological activity of babassu mesocarp extract (EE).* Material and Methods.* The* in vitro* antimicrobial activity was evaluated by disk diffusion assay and by determination of the minimum inhibitory concentration (MIC) to* Escherichia coli*,* Pseudomonas aeruginosa*,* Enterococcus faecalis*,* Staphylococcus aureus,* and methicillin-resistant* Staphylococcus aureus* (MRSA). The flavonoids and phenolic acids content were determined by chromatography. The* in vivo* assays were performed in Swiss mice submitted to sepsis by cecal ligation and puncture (CLP). The mice received EE subcutaneously (125 or 250 mg/Kg), 6 hours after the CLP. The number of lymphoid cells was quantified and the cytokines production was determined by ELISA after 12 h.* Results.* EE was effective as antimicrobial to* E. faecalis*,* S. aureus*, and MRSA. EE is rich in phenolic acids, a class of compounds with antimicrobial and immunological activity. An increased survival can be observed in those groups, possibly due to a significant inhibition of TNF-*α* and IL-6.* Conclusions.* The EE showed specific antimicrobial activity* in vitro* and an important antiseptic effect* in vivo* possibly due to the antimicrobial and immunomodulatory activity.

## 1. Introduction

Babassu (*Attalea speciosa* syn* Orbignya phalerata* Mart.) is the generic name of Brazilian native oleaginous palm trees from Arecaceae family. The mesocarp is obtained from the fruits and dried and ground into a powder, called babassu mesocarp flour. This product is widely commercialized either as a food supplement for adults and children or as medicine in the treatment of inflammatory and infectious diseases [1, 2].

A number of biological activities have been attributed to babassu mesocarp, such as anti-inflammatory [3], healing [4], antitumor [5, 6], antithrombotic [7], and antimicrobial properties [8]. In addition, the mesocarp is able to induce the release of hydrogen peroxide by macrophages suggesting an immunological effect on macrophage activation [9] and acts on cytokine production indicating an immunomodulatory effect [10].

Sepsis is a complex syndrome and still continues to be a major cause of morbidity and mortality among critically ill patients and at the intensive care units worldwide [11]. The significant morbidity and mortality associated with sepsis have continued to be powerful incentives for attempts to develop novel therapeutic strategies for this disease [12, 13]. The initial control of infection is very important to avoid the development of sepsis. The progression from a local to a systemic inflammatory and infective response is the result of the activation of circulating cells that release proinflammatory cytokines, such as interleukin 1 (IL-1), TNF-*α*, IL-6, and IL-8, into the circulation [14]. An increased production of chemokines is responsible for the recruitment of leukocytes to the inflammatory focus. Additionally, the release of anti-inflammatory cytokines seems to counterbalance the actions of proinflammatory mediators, either by reducing the synthesis and release of these mediators or by antagonizing their effects [15]. The goal of these mechanisms is to increase microbicidal activity and to control infection and systemic inflammatory response [16–18].

Antibiotic drugs generally interfere with the infection but not with the inflammatory response, a fact that might explain the high mortality rate observed in patients with septic shock [19, 20].

The progressive resistance of pathogenic microorganisms to multiple drugs [21–23] has encouraged the search for new agents, especially those derived from natural products. Therefore, the objective of the present study was to evaluate the* in vitro* and* in vivo* antimicrobial activity of babassu mesocarp extract in mice with sepsis by cecal ligation and puncture (CLP).

## 2. Material and Methods

### 2.1. Plant Material

All assays were carried out using flour prepared from* Attalea speciosa* fruits in our laboratory. The fruits were collected in Pedreiras, Maranhão, Brazil. A voucher specimen (number 1135/SLS017213) has been deposited at the Ático Seabra Herbarium, Federal University of Maranhão. Mesocarp, obtained manually, was dried at 45°C, 24 h, and ground to obtain the mesocarp flour.

### 2.2. Preparation of the Babassu Ethanol Extract (EE)

The babassu mesocarp flour (500 g) was air-dried at room temperature, powdered, and extracted in 2000 mL ethanol PA (Merck, Brazil), for 72 h. The extract was filtered and concentrated under low pressure at 24°C. The extract obtained was stored (10°C) prior to antimicrobial studies. The final yield was 7.9% (w/w).

### 2.3. Chemical Screening

The analysis of polyphenols and flavonoids was performed accordingly as previously described [24]. The total concentration of polyphenols was determined by Folin-Ciocalteu method using gallic acid as standard. The extract samples were serially diluted to a final volume of 2 mL. To each dilution 300 *μ*L of sodium carbonate (1.9 M) and 100 *μ*L of Folin were added. The solutions were incubated during one hour in the dark and the absorbances were measured at 760 nm. The content of total phenolic compounds was determined by the following formula: Abs ×* f* × dilution, considering* f* as the calibration factor to gallic acid [25].

The flavonoid concentration was measured with aluminum chloride at 425 nm using quercetin as standard. All samples were tested in triplicate. The phenolic acids concentration was determined from the difference between the flavonoids and total phenol concentration [26]. All results were expressed as similarity index (%)

### 2.4. *In Vitro* Assay to Antibacterial Activity

The dry EE was dissolved in phosphate-buffered saline, pH 7.2, to a final concentration of 500 mg/mL, sterilized by filtration (0.22 *µ*m). The reference strains were used for the* in vitro* antibacterial assay:* Escherichia coli* (ATCC 25922),* Pseudomonas aeruginosa* (ATCC 27853),* Enterococcus faecalis* (ATCC 29212), and* Staphylococcus aureus* (ATCC 25923). The antibacterial activity of EE was also evaluated against a strain of methicillin-resistant* Staphylococcus aureus* (MRSA) isolated from tracheal secretion from patients, which was susceptible to vancomycin and resistant to cefoxitin, cefazolin, clindamycin, erythromycin, ciprofloxacin, gentamicin, and tetracycline.

#### 2.4.1. Disk Diffusion Method (Kirby-Bauer)

The bacterial inoculum was adjusted to final concentration of 1.5 × 10^8^ CFU/mL (0.5, McFarland) and seeded onto a Mueller-Hinton agar plate. Sterile filter paper disks (6 mm) were placed on the plate and impregnated with 10 *µ*L of the EE at concentrations of 250 or 500 mg/mL. Disks impregnated with oxacillin (1 *µ*g) and cefoxitin (30 *µ*g) were used as positive controls. The plates were incubated at 35°C for 24 h [27].

#### 2.4.2. Determination of the Minimum Inhibitory Concentration (MIC) of EE

For the determination of MIC, a serial dilution of EE ranging from 500 to 0.9 mg/mL was added to tubes containing broth cultures of each strain, prepared in brain heart infusion [BHI (1.5 × 10^8^ UFC/mL, 0.5 on McFarland scale)], and incubated at 35°C, for 24 h. Tubes containing only BHI plus bacteria were used as positive controls and those ones containing BHI plus EE were considered as negative controls. The MIC is defined as the lowest concentration of the EE at which the microorganism tested does not demonstrate visible growth [28].

### 2.5. *In Vivo* Antimicrobial Activity: Sepsis

#### 2.5.1. Animals

Female Swiss mice weighing 25 ± 5 g were obtained from the Central Animal House of the Federal University of Maranhão.

#### 2.5.2. Sepsis Induction

Polymicrobial sepsis was induced by cecal ligation and puncture (CLP). Briefly, following anesthesia with sodium pentobarbital (50–65 mg/Kg, by intraperitoneal route i.p.), a small mid-abdominal incision was made and the cecum was exposed. A distended portion of the cecum just distal to the ileocecal valve was isolated and ligated with a silk suture in a manner not to disrupt bowel continuity. The ligated portion of the cecum was punctured eight times with an 18-gauge hypodermic needle. The abdomen was then closed in two layers and the animals were allowed to recover [15, 29].

#### 2.5.3. Experimental Design

The animals were randomly assigned to the experimental groups. Sham: the cecum was not perforated and the mice were not treated. The other 3 groups were given subcutaneously NaCl solution (CLP), EE 125 mg/Kg (EE125), or EE 250 mg/Kg (EE250). Animals were cared for in accordance with the guidelines of the Brazilian College of Animal Experimentation and the experimental protocol was approved by the Ethics Committee (protocol 23115011476/2007-50).

To evaluate the lifespan, the number of remaining animals was recorded every 12 h until the 5th day.

#### 2.5.4. Colony-Forming Units (CFU)

Bacterial counts were performed on aseptically obtained peritoneal fluid. At 12 h after CLP, mice were sacrificed and the skin of abdomen was cut open in the midline without injury to the muscle. Sterile phosphate-buffered saline (PBS) (2 mL) was injected into and aspirated out of the peritoneal cavities. Samples were serially diluted in PBS and cultured on Mueller-Hinton agar dishes (Difco Laboratories, Detroit). Colony-forming units were counted after overnight incubation at 37°C. The results were expressed as log_10_ of the number of colony-forming units per peritoneal cavity.

#### 2.5.5. Cytokines Assay

Serum TNF-*α*, IFN-*γ*, and IL-6 were measured by ELISA method in accordance with the manufacturer's instructions (eBiosciences, USA).

### 2.6. Statistical Analysis

All data were expressed as mean ± standard error (*X*  ± SE). Statistical significance was determined using ANOVA followed by Newman-Keuls test, Student's* t*-test. Kaplan-Meier curve and the log-rank statistical test were applied to compare the curves and for the evaluation of lifespan. Values with* p* < 0.05 were considered significant.

## 3. Results

### 3.1. The* In Vitro* Antimicrobial Activity of EE

The EE at the two tested concentrations inhibited the growth of* Enterococcus faecalis* (ATCC 29212),* Staphylococcus aureus* (ATCC 25923), and the MRSA strains. On the other hand, the same concentrations of EE have no effect on* Escherichia coli* (ATCC 25922) or* Pseudomonas aeruginosa* (ATCC 27853) strains (Table 1).

The MIC was determined only for the effective EE doses and bacteria strains. The MIC was 31.2 mg/mL for* S. aureus* (ATCC 25923) and MRSA and 7.8 mg/mL for* E. faecalis* (ATCC 29212). The highest concentration of EE (500 mg/mL) completely inhibited the growth of* S. aureus* (ATCC 25923), MRSA, and* E. faecalis* (ATCC 29212) (Table 2).

### 3.2. Chemical Composition of the EE

The predominance of phenolic compounds was detected. The extract contained 56% total polyphenols, including 55% phenolic acids and 1% flavonoids.

### 3.3. Effect of Babassu Mesocarp on Survival in CLP-Induced Mice Sepsis

**Figure 1:**
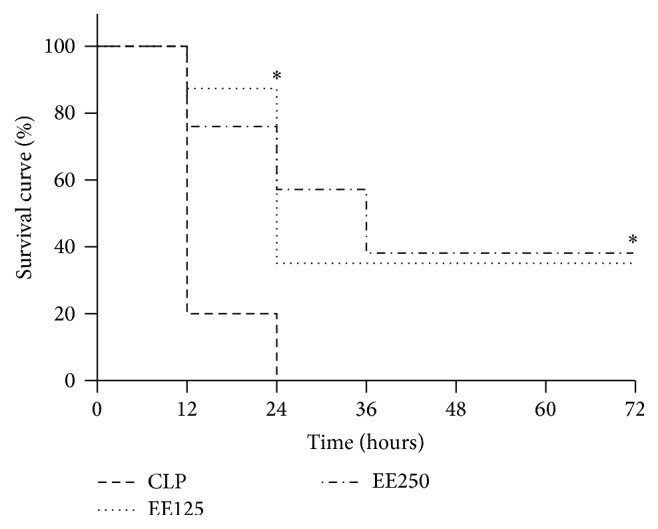
Effect of treatment with ethanolic extract (EE) of babassu mesocarp on the survival of mice with lethal sepsis induced by cecal ligation and puncture (CLP). The animals were treated with EE at doses of 125 mg/Kg (EE125) or 250 mg/Kg (EE250) 6 h after the induction of sepsis by cecal ligation and puncture and compared to animals that have received saline (CLP). The animals were examined at intervals of 12 h until day 10. The results are expressed as mean ± SEM (5 animals/group). (*∗*) *p* < 0,05 in comparison to the CLP group.

As shown in Figure 1, the survival in all groups submitted to sepsis was 100%, after surgery (T0). As shown in Figure 1 at the control group (CLP) the mortality was 80%, 12 hours after sepsis induction and, after 24 h, all animals of this group were dead. The onset of death was markedly delayed in mice that have received EE treatment. The survival in EE125 and EE250 groups was, respectively, 90% and 80%, after 12 hours and 40% and 60%, 24 h after the sepsis induction. In the EE125 group, survival remained constant (40%) until 10th day. In the EE250 group the survival rate only decreased to 40% after 36 h and remained unchanged thereafter.

### 3.4. Effect of EE on Cell Distribution on Peritoneum and Lymphoid Organs

**Figure 2:**
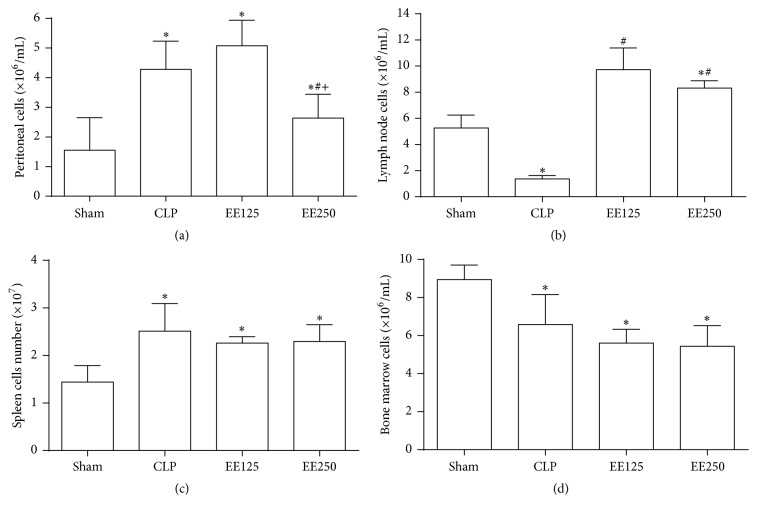
Effect of treatment with ethanolic extract (EE) of babassu mesocarp on the number of lymphoid cells. The animals were treated with EE at doses of 125 (EE125) or 250 mg/Kg (EE250) 6 h after the induction of sepsis by cecal ligation and puncture, sacrificed 12 h after the procedure, and compared to the untreated animals without (Sham) or with sepsis (CLP). The number of cells in the peritoneum (a), lymph nodes (b), bone marrow (c), and spleen (d) was quantified. The results are expressed as mean ± SEM (5 animals/group). (*∗*) *p* < 0,05 in comparison to the Sham group; (#) *p* < 0,05 in comparison to CLP group; and (+) *p* < 0,05 in comparison to EE250.

Sepsis by cecal perforation frequently induces an expressive increase in cell migration to the peritoneal cavity, as it was shown by the comparison between the group without sepsis (Sham) and group CLP, but the treatment with 250 mg/Kg of EE inhibited this cellular influx, in comparison to the other groups with sepsis (CLP and EE125) (Figure 2(a)). On the other hand, the EE treatment, irrespective of the dose, significantly increased the number of mesenteric lymph node cells, when compared to the cell numbers found in untreated groups without (Sham) or with sepsis (CLP) (Figure 2(b)). The treatment with EE has no effect on the number of splenocytes (Figure 2(c)) and bone marrow cells (Figure 2(d)).

### 3.5. Cytokine Production

**Figure 3:**
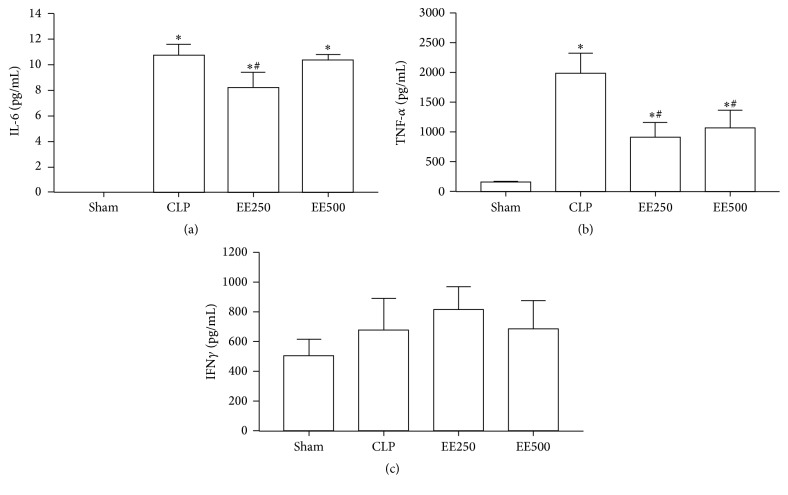
Effect of treatment with ethanolic extract (EE) of babassu mesocarp on the production of cytokines. The animals were treated with EE at doses of 125 (EE125) or 250 mg/Kg (EE250) 6 h after the induction of sepsis by cecal ligation and puncture and compared to the untreated group also submitted to the inductions of sepsis (CLP) or not (Sham). The animals were sacrificed 12 h after the procedure and serum was obtained for the measurement of IL-6 (a), TNF-*α* (b), and IFN-*γ* (c) by ELISA assay. The results are expressed as mean ± SEM (5 animals/group). (*∗*) *p* < 0,05 in comparison to the SHAM group and (#) *p* < 0,05 in comparison to the CLP group.

Treatment with EE inhibited the production of TNF-*α*, irrespective of the dose. In addition, treatment with EE125, but not with EE250, inhibited the production of IL-6. The production of IFN-*γ* was not affected by treatment with the extract (Figure 3).

## 4. Discussion

The medicinal potential of babassu (*Attalea speciosa*) has been recognized based on preclinical trials showing its biological activity. Phytochemical screening demonstrated a predominance of phenolic acids. Previous chemical studies using* O. phalerata* extracts also identified the presence of triterpenes, glycosylated triterpenes, tannins, sugars, saponins, and steroids [26].

The EE showed an effective antimicrobial* in vitro* activity against the Gram-positive bacteria* E. faecalis*,* S. aureus,* and MRSA. However, no activity was observed against* E. coli* and* P. aeruginosa*, which indicates a selective and specific antibacterial action of EE.

The MIC and minimum bactericidal concentration (MBC) were only determined for strains against which antimicrobial activity was observed in the disk diffusion assay. A higher MIC was observed for* E. faecalis*, although similar MBC were obtained for* E. faecalis*,* S. aureus,* and the hospital strain of* S. aureus* MRSA. The antimicrobial activity to Gram-positive bacteria for other vegetal extracts obtained from native and exotic species of the Brazilian flora was described previously [30–32]. The antimicrobial activity for* S. aureus* and MRSA was also reported previously [8], but the antimicrobial activity of babassu mesocarp to* E. faecalis* was not reported before.

*E. faecalis* is part of the commensal Gram-positive microbial flora of the gastrointestinal tract of humans and other mammals [33]. This microorganism is associated with nosocomial infections and may cause endocarditis and urinary infections. These bacteria are generally resistant to a wide variety of antibiotics, a fact that contributes to their high pathogenicity [34, 35].

Antimicrobial compounds of plant origin with a restricted action against specific bacteria species and/or strains are desired not only due to their efficacy in the infection control, but also because they permit the maintenance of the normal microbiota. One important finding of this study was the sensitivity of the hospital strain of MRSA to the babassu mesocarp extract, since therapeutic options for patients infected with MRSA are limited. MRSA strains are always resistant to all cephalosporins, including fourth-generation drugs, as well as to carbapenems, irrespective of the result of the antibiogram [36]. The glycopeptide antibiotics vancomycin and teicoplanin are often the only choice for the treatment of infections caused by these microorganisms.

An increasing resistance of* E*.* faecalis* to traditional antibiotics has been frequently reported in some Brazilian hospitals [37]. In addition, the growing resistance of these strains to vancomycin [37, 38] has encouraged studies aimed at the discovery of new therapeutic agents that act rapidly on the control of these microorganisms.

The presence of phenolic acids in the extract suggests a direct relationship with its antimicrobial activity since an antimicrobial action of these compounds has been attributed to their ability to form complexes with extracellular polysaccharides and proteins, rupturing the bacterial cell wall and inhibiting the enzymatic systems responsible for the synthesis of cell wall components [11, 39]. Since the pharmacological action of a plant species employed as a phytotherapeutic agent involves the interaction between its different chemical components [40], the antimicrobial activity of EE observed in this study may also be attributed to the synergistic action of phenolic compounds, specifically phenolic acids, and flavonoids present in the extract.

In view of the restricted number of active products available for the control of MRSA and of the growing resistance of* E. faecalis*, babassu mesocarp might be used as a potential target for prospecting bioactive compounds with controlled antibiotic action.

Due to this antibacterial action, the effect of EE treatment on* in vivo* bacterial systemic infection was evaluated using the CLP model which mimics the events that occur in sepsis in humans in terms of both surgical trauma and the involved microbiota [15, 29].

The CLP model reproduces a type of infection mainly caused by* Escherichia coli*, Gram-negative bacteria [15], but evaluates the possible coexistence of other bacteria colonizing the abdominal cavity, in this case Gram-positive bacteria. CLP alone triggers a series of proinflammatory events that are characteristic of a systemic inflammatory response, including cell and tissue injury, neutrophil migration, reduction in the number of cells in adjacent lymph nodes and spleen, and increased production of proinflammatory cytokines and other mediators. This set of events eventually results in multiple organ failure and death.

Treatment with EE was initiated 6 h after the induction of sepsis to evaluate the therapeutic effect of the extract. The results showed that treatment with the babassu extract did not affect bacterial counts but increased the survival of the animals and immunoregulated the proinflammatory cytokine production. Similar results were previously described by Maciel et al. [29] studying the effect of* Syzygium jambolanum* in sepsis. They showed an increased survival in lethal sepsis more associated with the anti-inflammatory effect than with the antibiotic activity of the extract.

Mice from EE250 group showed inhibition of peritoneal cell migration and a lower production of TNF-*α* and IL-6 when compared to the control group. IL-6, TNF-*α*, and IL-8 exert an inflammatory action and an increased level of these cytokines is frequently found in septic shock caused by Gram-negative bacteria as observed in the CLP model [18, 19, 21, 36].

Proinflammatory cytokines such as TNF-*α*, IL-6, IL-1, and IFN-*γ* play an important role during the course of sepsis, interfering with the prognosis, progression, and intensity of tissue damage, and are associated with the aggravation and lethality of sepsis [11, 18]. TNF-*α* is the first cytokine in the blood circulation in human sepsis and promotes leukocytes recruitment to the inflammatory focus. Additionally, TNF-*α* is associated with an increase in the production of chemokines and adhesion molecules that are involved in the recruitment, proliferation, and survival of cells at sites of injury [19]. Taken together, these data suggest that the lower production of TNF-*α* observed in animals treated with EE250 might be associated with the inhibition of cell migration to the peritoneum. This hypothesis is supported by the increased number of cells detected on adjacent lymph nodes observed in the EE250 group.

Babassu mesocarp flour has a potent activating effect on macrophages as shown previously [3]. The treatment with EE125 was more effective in decreasing the IL-6 production and increased the cellular influx to the peritoneal cavity, in contrast to the treatment with the high dose EE250 that had no effect on IL-6 production, but efficiently reduced the cellular influx to peritoneal cavity. The differences between the doses can be related to the efficacy of the compounds present in babassu mesocarp to regulate the IL-6 production and the expression of receptors that can be associated with cellular migration [41, 42] and with the EE effect on the outcome and survival in experimental sepsis.

Taken together, the present results show that the efficacy of EE in increasing the lifespan in mice with sepsis by CLP is related to an immunomodulatory effect on the inflammatory process, mediated by cytokines. The immunomodulatory effect of babassu mesocarp on inflammation has been reported in other studies [3, 9, 10]. The present results confirm this property and also indicate that this action is possibly related to the capacity of compounds present in the extract to inhibit the production of TNF-*α* and IL-6 cytokines.

In conclusion the present study showed that the EE has a relevant and selective bacteriostatic action* in vitro* against the Gram-positive bacteria* Staphylococcus aureus* and* Enterococcus faecalis*, but not against the Gram-negative bacteria* Escherichia coli* and* Pseudomonas aeruginosa*. Besides the EE increases the survival of animals submitted to the lethal sepsis, activity possibly related to a decrease in TNF-*α* and IL-6 production and a consequent inhibition of systemic inflammation.

## Figures and Tables

**Table 1:** Antimicrobial activity of babassu mesocarp ethanolic extract evaluated by disk diffusion assay.

Bacterial strains	Zones of inhibition (mm)	
	EE250^a^	EE500
*Enterococcus faecalis *(ATCC 29212)	12.4 ± 0.2^b^	14.4 ± 0.4
*Staphylococcus aureus *(ATCC 25923)	15.0 ± 0.3	18.5 ± 0.9
MRSA (hospital strain)	15.3 ± 0.3	17.4 ± 0.3
*Escherichia coli (ATCC 25922)*	0	0
*Pseudomonas aeruginosa*	0	0

^a^EE babassu mesocarp ethanolic extract at concentrations of 250 and 500 mg/mL.

^b^The diameters of zones of inhibition (mm) are expressed as mean ± SD (*n* = 3); a diameter less than 7 mm was considered inactive; and the diameters of zones of inhibition (mm) are expressed as mean ± SD (*n* = 3).

**Table 2:** Minimum inhibitory concentration of babassu mesocarp ethanolic extract.

Bacterial strains	MIC^a^ (mg/mL)
*Enterococcus faecalis *(ATCC 29212)	7.8^b^
*Staphylococcus aureus *(ATCC 25923)	32.1
MRSA (hospital strain)	32.1

^a^MIC: minimum inhibitory concentration.

^b^Values represent the mean of triplicates (*n* = 3).
